# Applications of Genetically Modified Immunobiotics with High Immunoregulatory Capacity for Treatment of Inflammatory Bowel Diseases

**DOI:** 10.3389/fimmu.2017.00022

**Published:** 2017-01-25

**Authors:** Suguru Shigemori, Takeshi Shimosato

**Affiliations:** ^1^Department of Bioscience and Food Production Science, Interdisciplinary Graduate School of Science and Technology, Shinshu University, Nagano, Japan; ^2^Japan Society for the Promotion of Science, Tokyo, Japan; ^3^Department of Interdisciplinary Genome Sciences and Cell Metabolism, Institute for Biomedical Sciences, Shinshu University, Nagano, Japan; ^4^Supramolecular Complexes Unit, Research Center for Fungal and Microbial Dynamism, Shinshu University, Nagano, Japan

**Keywords:** probiotics, immunobiotics, IBD, gmLAB, gm-immunobiotics

## Abstract

Inflammatory bowel diseases (IBDs), including ulcerative colitis and Crohn’s disease, are chronic inflammatory diseases characterized by dysregulated immune responses of the gastrointestinal tract. In recent years, the incidence of IBDs has increased in developed nations, but their prophylaxis/treatment is not yet established. Site-directed delivery of molecules showing anti-inflammatory properties using genetically modified (gm)-probiotics shows promise as a new strategy for the prevention and treatment of IBD. Advantages of gm-probiotics include (1) the ability to use bacteria as a delivery vehicle, enabling safe and long-term use by humans, (2) decreased risks of side effects, and (3) reduced costs. The intestinal delivery of anti-inflammatory proteins such as cytokines and enzymes using *Lactococcus lactis* has been shown to regulate host intestinal homeostasis depending on the delivered protein-specific machinery. Additionally, clinical experience using interleukin 10-secreting *Lc. lactis* has been shown to be safe and to facilitate biological containment in IBD therapy. On the other hand, some preclinical studies have demonstrated that gm-strains of immunobiotics (probiotic strains able to beneficially regulate the mucosal immunity) provide beneficial effects on intestinal inflammation as a result of the synergy between the immunoregulatory effects of the bacterium itself and the anti-inflammatory effects of the delivered recombinant proteins. In this review, we discuss the rapid progression in the development of strategies for the prophylaxis and treatment of IBD using gm-probiotics that exhibit immune regulation effects (gm-immunobiotics). In particular, we discuss the type of strains used as delivery agents.

## Introduction

Inflammatory bowel disease (IBD) is a chronic inflammatory disease that occurs in the gastrointestinal tract (GIT); IBDs are largely classified as ulcerative colitis (UC) and Crohn’s disease (CD). There has been an increase in the number of cases of IBD in recent years, mainly in Western countries (1). IBD causes inflammatory obstruction of the GIT, resulting in symptoms such as stomach cramps, pain, diarrhea, constipation, and vomiting over an extended period of time. These symptoms cause considerable reduction in quality of life. While IBD is not a direct cause of mortality, the disease can increase the risk of colorectal cancer (2). The precise etiology of IBD has yet to be clarified, but causal factors are thought to include the environment, genetics, and microorganisms (3). The chronic inflammation seen in IBD is characterized by dysregulated immune response of the host as a result of marked changes in the intestinal environment (3). Consequently, favorable regulation of the compromised immune homeostasis is effective in the prognosis and treatment of IBD. Corticosteroids, thiopurines, and anti-tumor necrosis factor (TNF) antibody (Ab), which exhibit immune-regulatory effects, can control IBD to a certain extent, and these treatments are widely used in clinical settings as therapeutic drugs (4). However, there are individual-specific differences in the effectiveness of these drugs, and there are also issues such as the possibility of serious side effects and high costs (4, 5).

**Figure 1:**
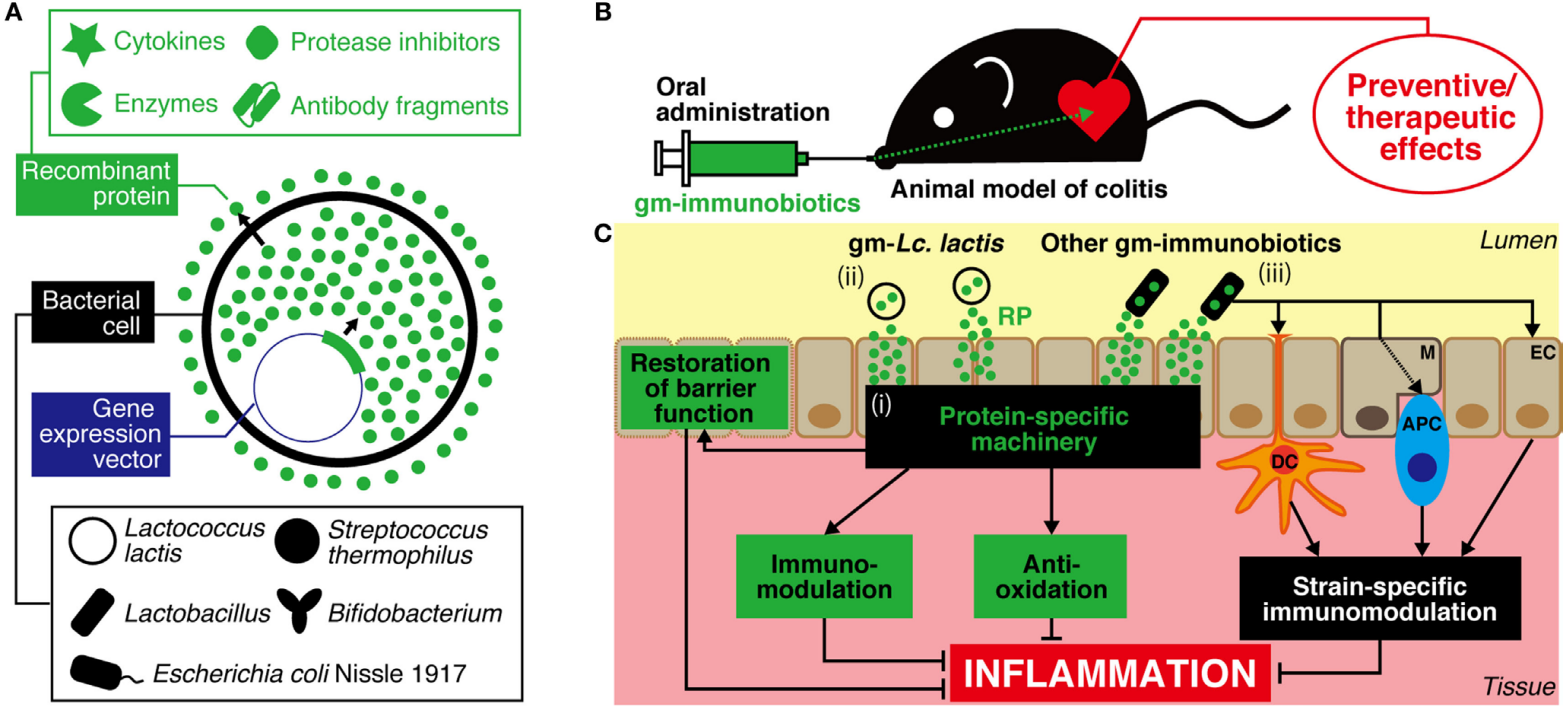
**A strategy for prevention and treatment of IBD using genetically modified (gm)-immunobiotics**. **(A)** Different bioactive proteins such as cytokines, enzymes, protease inhibitors, and antibody fragments can be produced/secreted by gm-strains. **(B)** After oral administration, viable cells of gm-immunobiotics transit through the gastric environment and reach the intestine. Then, gm-immunobiotics provide preventive/therapeutic effects against experimental colitis in animal as a result of the exertion of anti-inflammatory effects *in situ*. **(C)** General mechanisms of action of gm-immunobiotics on anti-inflammatory effects in the intestine. Physiologically meaningful amounts of recombinant proteins are yielded by gm-immunobiotics *via* secretion or cell lysis, and exert host anti-inflammatory effects through a protein-specific machinery including immunomodulation, anti-oxidation, and restoration of epithelial barrier functions (i). *Lactococcus* (*Lc*.) *lactis* has been most widely used as a safe and effective vector in this strategy (ii). *Lc. lactis* has little or no effect on either the improvement or aggravation of the intestinal inflammation and does not colonize the intestine. Other gm-immunobiotics (including some strains of *Lactobacillus, Bifidobacterium*, and *Streptococcus salivarius* subsp. *thermophilus*, and *Escherichia coli* Nissle 1917) provide beneficial effects on intestinal inflammation as a result of the synergy between the immunoregulatory effects of the bacterium itself and the anti-inflammatory effects of the delivered recombinant proteins (iii). Immunobiotics interact with pattern recognition receptors of host epithelial cells and antigen-presenting cells such as dendritic cells and macrophages to exert strain-specific immunomodulatory effects. Some strains of immunobiotics may colonize the intestine. IBD, inflammatory bowel disease; RP, recombinant protein; EC, epithelial cell; M, microfold cell; DC, dendritic cell; APC, antigen-presenting cell.

There is currently a great deal of interest in the use of probiotics that have been genetically modified (gm) to produce proteins with IBD therapeutic potential as novel drug substitutes. Probiotics, defined as “live microorganisms that, when administrated in adequate amounts, confer a health benefit on the host” (6), have been reported to attenuate inflammation in the host GIT through immune system regulation, strengthening of barrier function, and improvement of the changed intestinal microbiota (7). Probiotics comprise primarily lactic acid bacteria (LAB) and bifidobacteria, and also include non-pathogenic *Escherichia coli*. Probiotics have been used in food for a long time, and many of the bacteria included in probiotics fall under the Generally Recognized As Safe assessment designated by the United States Food and Drug Administration and meet the Qualified Presumption of Safety designation of the European Food Safety Authority. Genetic modification technology has undergone considerable advances in recent years, and *Lactococcus* (*Lc*.) *lactis* in particular has been established as an efficient expression system for recombinant proteins (RPs) (8) (Figure 1A). Thus, probiotics, which have excellent safety and health advantages, are likely to be very useful as producers of IBD therapeutic proteins and as agents for delivering such proteins to the GIT (Figure 1B). gm-Probiotics that produce or secrete various different anti-inflammatory proteins have been constructed in recent years, and their anti-inflammatory effectiveness when administered orally has been verified using *in vivo* experiments in animal models of IBD (9, 10) (Table 1; Table S1 in Supplementary Material). In this context, it is important to note that the delivery of IBD therapeutic proteins to the GIT using gm-probiotics is expected (1) to allow the therapeutic protein to act locally, with greater effectiveness and decreased risk of medical error or side effects compared to conventional systemic administration of the molecule by injection, and (2) to be considerably cheaper than refined drugs (10, 11). It is of particular interest that many of the molecules selected as anti-inflammatory proteins target the host immune system. Many studies to date have used *Lc. lactis* as a model strain, but methods using lactobacilli, bifidobacteria, streptococci, and *E. coli* Nissle 1917 (EcN), bacteria that have more beneficial health effects than *Lc. lactis*, as delivery agents have been attempted in recent years (Figure 1A). Many of these studies (12–26) employ bacteria that have been termed “immunobiotics,” which have been defined as probiotic strains that are able to beneficially regulate mucosal immunity (27, 28). Immunobiotics are recognized by the pattern recognition receptors of epithelial and antigen-presenting cells such as dendritic cells and macrophages, and these immunobiotics are known to beneficially regulate innate and adoptive immune responses (Figure 1C); there have been tremendous advances in the clarification of strain-specific immune regulation functions at the cellular and molecular levels (28–32).

**Table 1:** **Selected preclinical evidence showing beneficial effects of gm-immunobiotics in treatment of gastrointestinal tract inflammation**.

Strains	Recombinant protein	Disease model	Outcome	Efficacy	Potential mechanisms	Reference
*Lc. lactis* MG1363/NZ9000	IL-10	mDAC, mTAC, m*IL-10*^−/−^	Reduction in MS, HS, and IM (MPO, Cox-2, SAA)	CC = WT/VC < Objects	Immunomodulation	(33–37)
			Modulation of P/AICy			

*Lc. lactis* MG1363	IL-27	mTTC, mDAC	Reduction in Mo, MS, and HS	CC = Systemic IL-27 = VC < Object	Immunomodulation	(38)
			Modulation of P/AICy and PTc	MG1363-IL-10 < Object		

*Lc. lactis* NZ9000	Elafin/SLPI	mDAC, mDCC, mTTC, hIEC	Reduction in MS, HS, CT, IIP, and IM (PL, MPO, PICy, PIL)	CC ≤ WT < NZ9000*-*IL-10/TGF-β < Objects	Reduction in elastolytic activity	(33, 39)

*Lc. lactis* NZ9000	HO-1	mDAC	Reduction in MS, HS, and CS	CC = VC < Object	Immunomodulation	(40)
			Modulation of P/AICy			

*Lb. casei* BL23	Cat/SOD	mDAC, mTAC	Reduction in MS, HS, and LMT	CC ≤ WT/VC < Objects	Reduction in oxidative stress	(15, 17, 18)
			Modulation of P/AICy		Immunomodulation	

*Lb. casei* BLS	α-MSH	mDAC	Reduction in Mo, MS, HS, CS, and IM (MPO, NF-κB)	CC ≤ WT < Object	Immunomodulation	(23)
			Modulation of P/AICy			

*S. thermophilus* CRL807	Cat/SOD	mTAC	Reduction in Mo, MS, HS, and LMT	CC < WT < Objects	Reduction in oxidative stress	(13)
			Modulation of CPIc		Immunomodulation	

*B. longum* NCC2705	IL-10	mDAC	Reduction in Mo, MS, HS, CS, and IM (MPO, NF-κB)	CC < WT/VC < Object	Immunomodulation	(21, 22)
			Modulation of PTc and P/AICy			

EcN	AvCys	mDAC, pPWD, hIEC	Reduction in MS, HS, CS, IIP, and IM (PIM, PICh, PICy)	CC ≤ WT < Object	Immunomodulation	(24)
			Increase in Treg, TER		Improvement of intestinal barrier function	

*Lc., Lactococcus; Lb., Lactobacillus; S. thermophilus, Streptococcus salivarius subsp. thermophilus; B., Bifidobacterium; EcN, Escherichia coli Nissle 1917; IL-10, interleukin 10; IL-27, interleukin 27; SLPI, secretory leukocyte protease inhibitor; HO-1, heme oxygenase-1; SOD, superoxide dismutase; Cat, catalase; α-MSH, α-melanocyte-stimulating hormone; AvCys, cystatin from Acanthocheilonema viteae; mDAC, murine dextran sulfate sodium-induced acute colitis; mTAC, murine 2,4,6-trinitrobenzene sulfonic acid-induced acute colitis; mIL-10^−^^/^^−^, spontaneous colitis in IL-10-deficient mice; mTTC, murine T-cell transfer-induced enterocolitis; mDCC, murine dextran sulfate sodium-induced chronic colitis; hIEC, human intestinal epithelial cells; pPWD, porcine post-weaning diarrhea; MS, macroscopic symptoms; HS, histological symptoms; IM, mediators of inflammation; MPO, myeloperoxidase activity; Cox-2, cyclooxygenase-2 activity; SAA, serum amyloid A; P/AICy, pro-/anti-inflammatory cytokines; Mo, mortality; PTc, phenotypes of T-cell; CT, colon thickening; IIP, intestinal epithelial permeability; PL, proteolytic activity; PICy, pro-inflammatory cytokines; PIL, pro-inflammatory leukocytes; CS, colon shortening; LMT, liver microbial translocation; NF-κB, nuclear factor-κB; CPIc, cytokine phenotypes of immune cells; PIM, pro-inflammatory macrophages; PICh, pro-inflammatory chemokines; Treg, regulatory T-cell; TER, transendothelial electrical resistance; CC, colitis control; WT, wild-type strain; VC, vector control; MG1363-IL-10, IL-10-secreting Lactococcus lactis MG1363; NZ9000-IL-10/TGF-β, IL-10- or TGF-β-secreting Lactococcus lactis NZ9000*.

In this review, we describe recent developments in preventive and therapeutic strategies for the treatment of IBD using gm-probiotics. In particular, our discussion focuses on gm-probiotics that exhibit immune regulation effects (gm-immunobiotics) and bacterial species that are used as protein delivery agents.

## *Lactococcus* *lactis*

*Lactococcus lactis* is a species of LAB used universally in cheese and other fermented dairy products. To date, *Lc. lactis* MG1363 (MG1363) and its derivatives have been widely used to produce RPs and as carriers for delivery to mucous membranes (Figure 1). *Lc. lactis* was the first LAB species to have its whole genome sequenced, and there exists a wealth of genetic data on this species (8, 41, 42). In addition, *Lc. lactis* genetic modification is straightforward, and there are a great number of useful gene expression systems for this organism (8). Furthermore, *Lc. lactis* is able to pass through the GIT alive but does not establish itself in the GIT and is easy to control pharmacokinetically (43, 44). It is important to note that *Lc. lactis* itself has little or no effect on either the improvement or aggravation of GIT inflammation in animals and humans and is therefore highly safe for use against IBD (14, 33–35, 38–40, 45–52) (http://ClinicalTrials.gov Identifier: NCT00729872). The research to date into gm-*Lc. lactis* has been compiled into a number of review articles (9–11, 53). In the present review, we will deal with a series of landmark studies that showed the usefulness and practicality of the present strategy, and we will examine the latest findings.

The strategy of reducing intestinal inflammation by using gm-probiotics for delivery of RPs to the GIT was first proposed in 2000 by Steidler et al. (35), who created a MG1363 strain that secreted interleukin (IL)-10 (LL-mIL10). IL-10 is a cytokine that plays a central role in the suppression of inflammation (54), and mutation of the endogenous gene has been shown to be involved in the onset of murine enterocolitis (55, 56) and infantile-onset IBD (57, 58). Steidler et al. showed that daily oral administration of LL-mIL10 resulted in a dramatic reduction of colitis onset and progression in a murine IBD model (35). Notably, the effective amount of IL-10 was 1/10,000th of the amount used in conventional systemic administration. This enhancement may be regarded as the greatest advantage of the present strategy. The reduction in the amount administered has also been demonstrated in the delivery systems of other RPs (38, 49, 50). Next, Steidler et al. constructed LL-Thy12, in which the thymidylate synthase gene (*thyA*) of the *Lc. lactis* genome was replaced by the human IL-10-encoding gene (59). The results of a phase 1 clinical study in CD patients confirmed the safety, biological containment, and significant therapeutic effect of LL-Thy12 (52). However, no statistically significant therapeutic effect was found in the subsequent phase 2a clinical study (http://ClinicalTrials.gov Identifier: NCT00729872). The authors suggested that the lack of therapeutic effect was due to low concentration of IL-10 in the intestine. Nonetheless, bearing in mind that this first clinical study using gm-LAB suggested the safety and usefulness of this delivery system, the results were remarkable.

IL-27 is an anti-inflammatory cytokine belonging to the IL-12 family, a group of molecules that has been shown to attenuate murine experimental colitis by suppressing the development of T helper 17 (Th17) cells (60). In addition, the involvement of low-expressing variants of the IL-27-encoding gene in early-onset IBD has been demonstrated (61). In 2014, Hanson et al. showed that daily oral administration of MG1363 that secretes IL-27 (LL-IL-27) almost completely cured murine T-cell transfer-induced enterocolitis and reduced the associated mortality rate (38). LL-IL-27 treatment caused a reduction in the level of inflammatory cytokines that had increased in the GIT as a result of enterocolitis and a reduction in the number of colitis pathogenic IL-17-producing T-cells. In addition, the results indicated that increased local production of IL-10 by LL-IL-27 in the GIT was effective in providing a therapeutic effect. It is important to note that oral administration of LL-IL-27 demonstrated a notably greater therapeutic effect than systemic administration of IL-27 or oral administration of IL-10-secreting MG1363.

In 2015, a study comparing *Lc. lactis* NZ9000 (NZ9000) that secreted serine protease inhibitors (elafin or secretory leukocyte protease inhibitor) to NZ9000 that secreted the anti-inflammatory cytokines IL-10 or transforming growth factor-β showed that the former significantly attenuated the symptoms of dextran sodium sulfate (DSS)-induced colitis (33). Prior to that study, Motta et al. showed that the expression of elafin was lower in IBD patients than in healthy people, and that this decreased expression correlated with the increased elastolytic activity of the colonic mucosa in IBD patients (39). Also, delivery of elafin to the GIT using a gm-NZ9000 resulted in marked improvement of acute and chronic colitis in murine models (39). Elafin-secreting NZ9000 restored the colonic elastolytic homeostasis that had broken down as a result of colitis, reduced the number of immune cells infiltrating the colon, and repaired the barrier function of the intestinal epithelium (39).

In 2015, we successfully constructed a gm-NZ9000 strain (designated NZ-HO) that secretes biologically active heme oxygenase-1 (HO-1). HO-1 is an enzyme that catalyzes heme catabolism *in vivo*. HO-1 is induced endogenously by stimuli such as inflammation or oxidative stress, and the enzyme exhibits anti-inflammatory and cytoprotective effects mediated by the generation of heme breakdown products (62, 63). We showed that daily oral administration of NZ-HO markedly attenuated the symptoms of DSS colitis (40). Interestingly, NZ-HO increased the production of IL-10, decreased inflammatory cell infiltration, and decreased expression of IL-6 and IL-1α in the colonic tissue of murine colitis models (40). In 2014, Zhang et al. showed that intraperitoneal injection of an HO-1 inducer-induced IL-10-producing regulatory T cells (Treg) (rather than IBD pathogenic Th17) by inhibiting IL-6/IL-6 receptor signaling, thus ameliorating DSS colitis (64). This result suggested that NZ-HO regulates the immune responses of the inflamed colon in a beneficial fashion to ameliorate DSS colitis.

In 2015, Aubry et al. found that preventive oral administration of MG1363 that secreted thymic stromal lymphopoietin caused a transient increase in the number of CD4^+^ CD25^+^ FoxP3^+^ Treg cells in the mesenteric lymph node and attenuated DSS colitis in mice (45). Quevrain et al. found that MG1363 that secreted an anti-inflammatory protein (MAM) isolated from a strain of *Faecalibacterium prausnitzii*, a species that is deficient in CD patients and alleviated dinitrobenzene sulfonic acid-induced colitis in mice (47). MAM-secreting MG1363 markedly reduced the production of pro-inflammatory cytokines (IL-17A and interferon-γ) in the colonic tissue of colitis mice (47).

IL-6 is an important pathogenic factor in various different inflammatory diseases, including IBD. By regulating the function and proliferation of T cells, IL-6 exacerbates GIT inflammation in IBD (65). In addition, studies using murine models of colitis and CD patients showed that inhibition of IL-6 signaling using antibodies improved the symptoms (66, 67). However, the cost of Ab drugs is very high. We therefore created a NZ9000 derivative that secretes a single-chain variable fragment Ab against IL-6 (IL6scFv) (68). Importantly, we showed that the recombinant IL6scFv produced by gm-NZ9000 is immunoreactive, as demonstrated by binding to IL-6 (68). Thus, IL6scFv-secreting NZ9000 is an attractive gm-LAB for research and development of a low-cost IBD therapeutic drug that can yield site-directed delivery of anti-IL-6 antibodies.

## *Lactobacillus* 

Bacteria of the genus *Lactobacillus*, which are classified as LAB, are the best-known type of probiotics. Several strains belonging to this genus are commensal bacteria that reside within the human GIT. To date, many preclinical studies have indicated that strains belonging to genus *Lactobacillus* regulate GIT inflammation in a favorable fashion through strain-specific, health-beneficial mechanisms (9). In addition, clinical research to date has shown that a probiotic mixture containing four species of *Lactobacillus* (VSL#3) and *Lactobacillus reuteri* ATCC 55730 exhibits benefits in the treatment of active UC (69–72). Bacteria belonging to genus *Lactobacillus* are used predominantly in probiotic formulations that are useful for the prevention and drug therapy of GIT-related diseases selected by the World Gastroenterology Organization (73).

In 2007, Rochat et al. showed that daily oral administration of *Lactobacillus casei* BL23 (BL23) attenuated murine DSS colitis (17). The same year, Foligne et al. demonstrated that BL23 induced an immune reaction with dominance of anti-inflammatory IL-10 over pro-inflammatory IL-12 in human peripheral blood mononuclear cells and reduced the symptoms of murine 2,4,6-trinitrobenzenesulfonic acid (TNBS) colitis (74). In 2010, Watterlot et al. orally administered superoxide dismutase (SOD)-producing and SOD-non-producing BL23 to mice and found that the former resulted in marked amelioration of DSS-induced histological damage to the colon, while the latter gave only slight amelioration (18). An excess of reactive oxygen species causes considerable tissue damage, which suggests a link to IBD development, and the use of antioxidative enzymes to eliminate reactive oxidative species is expected to have potential as an IBD treatment strategy (75). Oral delivery of SOD using gm-LAB has actually been shown to reduce colitis in rodents (12, 14). In 2011, LeBlanc et al. orally administered BL23 that produced an antioxidative enzyme (SOD or catalase) to mice, and their results showed that the mortality rate, weight loss, histological colon damage, and liver microbial translocation induced by TNBS administration were markedly reduced (15). However, in the studies performed by Watterlot et al. (18) and LeBlanc et al. (15), wild-type (WT) BL23 had only mild anti-inflammatory properties and did not induce marked IL-10 production in colon tissue, indicating that the amelioration effects on murine colon inflammation are limited. In 2014, Hou et al. showed that oral administration of SOD-producing *Lactobacillus fermentum* I5007 (I5007) improved lipid peroxidation and immune parameters in the colon, thus ameliorating murine TNBS colitis (26). A partial, but significant, improvement effect was also observed with WT-I5007. I5007 was isolated from healthy porcine intestinal mucosa and has been used as a growth stimulator for livestock. The above series of studies proposed a novel IBD preventive strategy combining the two different intestinal inflammation amelioration mechanisms: the immunobiotic effects of lactobacilli and the antioxidative effects of delivered proteins (Figure 1C).

In 2008, α-melanocyte-stimulating hormone (α-MSH)-secreting *Lb. casei* BLS (BLS) was created (23). α-MSH is a neuropeptide with immunosuppressant effects that has been reported to exhibit anti-inflammatory effects in animal models of various diseases, including IBD (76). Orally administered gm-BLS shows curative effects for the symptoms of murine DSS colitis (23). This improvement involves decreased secretion of inflammatory cytokines (TNF-α, IL-1β, and IL-6) and increased secretion of immune-regulatory cytokines (IL-4 and IL-10) in *ex vivo* cultures of colonic tissue (23). It is interesting to note that gm-BLS brought about considerable improvement in a number of parameters when compared to the WT strain (23).

## *Streptococcus salivarius* subsp. *thermophilus* (*S. thermophilus*)

*Streptococcus thermophilus* is a LAB that has traditionally been used as a yogurt starter. Preclinical studies to date have clarified the roles of specific *S. thermophilus* strains as immunobiotics (77–82). For example, Ogita et al. showed that *S. thermophilus* ST28 (ST28) derived from milk regulated IL-17 production in murine splenocytes in Th17-skewed conditions by induction of counteracting interferon-γ (82). Moreover, oral administration of ST28 to mice markedly decreased DSS-induced intestinal lesions, and this treatment markedly decreased IL-17 secretion and the frequency of accumulation of Th17, the numbers of which had increased in the lamina propria as a result of DSS (81). *S. thermophilus* is a component of a probiotic mixture agent (VSL#3) that has been found to be effective for induction and maintenance of remission in UC and prevention and maintenance of remission in pouchitis (73). It is interesting to note that several *S. thermophilus* strains are known to be autolytic, a useful trait for strains used as gm-immunobiotics (83).

In 2014, an immunobiotic strain, *S. thermophilus* CRL807 (CRL807), which exhibits immunosuppressant action *in vitro* and *in vivo*, was selected from a mixed yogurt starter; CRL807’s usefulness as a delivery agent for SOD and catalase then was investigated (13). CRL807 significantly increased the ratios of IL-10:inflammatory cytokine (IL-12, IL-17, or interferon-γ) in human peripheral blood mononuclear cells and the digestive tract of healthy mice. Oral administration of antioxidative enzyme-producing gm-CRL807 and WT-CRL807 to mice markedly potentiated the ratio of IL-10-positive:IL-17-positive cells, a ratio that had been reduced by TNBS administration, and provided amelioration of colitis. Notably, administration of either or both SOD-producing and catalase-producing CRL807 improved antioxidative enzyme activity in the colon, demonstrating greater anti-inflammatory action than WT-CRL807 administration. Experimental long-term (30-day) oral administration of gm-CRL807 and WT-CRL807 in healthy mice showed the safety of CRL807 (84).

## *Bifidobacterium* 

The genus *Bifidobacterium* comprises indigenous bacteria that make up the intestinal flora and in particular are present in significant numbers in healthy infants. In IBD patients, on the other hand, it is known that there is a decreased number of *Bifidobacterium* and an increase in pro-inflammatory *E. coli* and *Bacteroides* in the intestinal mucosa (85–89). Preclinical studies to date have shown that various strains of genus *Bifidobacterium* bring about beneficial effects in the prevention and treatment of colitis, mediated by different effects [immunoregulation effects (90, 91), improvement of the barrier function of intestinal epithelium (92, 93), and improvement of the intestinal flora (94, 95)]. It is interesting that *Bifidobacterium longum* subsp. *infantis* 35624 has been shown to selectively drive specialization of FoxP3^+^ Treg cells and/or induce IL-10 production in animal disease models and in humans (96–99). In addition, clinical studies of patients with UC and other inflammatory diseases showed that, compared to placebo, oral administration of this immunobiotic strain resulted in a marked decrease in the level of plasma C-type protein, an inflammatory biomarker that increases with the disease (100). It has also been shown that the symptoms of UC patients are ameliorated by a single Bifidobacteria strain (101), probiotic mixtures that include Bifidobacteria (69, 71, 72, 102, 103), and symbiotics (probiotic/prebiotic mixtures) in which Bifidobacteria is the main constituent (104–106).

In 2011, an immunobiotic strain, *B. longum* NCC2705 (NCC2705), was engineered to secrete biologically active IL-10, and the strain’s curative effects in DSS colitis were investigated (21). Improvement of the symptoms of DSS colitis (aggravation of gross symptoms, colon shortening, histopathological changes accompanying tissue damage, and myeloperoxidase activation) was observed with oral administration of WT-NCC2705 alone. Considerable improvement was found with IL-10-secreting gmNCC2705 when compared to WT-NCC2705 treatment (21). In addition, this study found that WT-NCC2705 and gm-NCC2705 reduced the expression of nuclear factor-κB and pro-inflammatory cytokines in the colon and the peripheral blood, and restored the proportion of CD4^+^ CD25^+^ FoxP3^+^ Treg cells (21). These effects were markedly stronger with gm-NCC2705. In 2015, Zhang et al. showed that the Treg/Th17 balance that had broken down as a result of DSS colitis was fully restored by gm-NCC2705 through the inhibition of two intracellular signaling pathways for Th17 induction (22). In 2016, the intestinal inflammation amelioration action of different strains of *B. longum* that produced human α-MSH was reported (19, 20). In the first of these reports, preventive daily oral administration of α-MSH-secreting *B. longum* HB15 (HB15) markedly reduced histopathological damage, increased myeloperoxidase activity, corrected an inflammatory/anti-inflammatory cytokine imbalance, and induced production of the pro-inflammatory factor nitrogen monoxide, overcoming effects caused by DSS colitis in rats. Administration of WT-HB15 improved all the parameters with the exception of nitrogen monoxide production, but to a considerably lower degree than that seen with the recombinant strain (19). In the second report, α-MSH-secreting *B. longum* HB25 (HB25) was created. Therapeutic daily oral administration of this recombinant strain markedly improved murine DSS colitis. Interestingly, no curative effects were observed from oral administration of the vector control strain (20). The two serial studies above indicated that immunobiotic Bifidobacteria that secrete proteins exhibiting immunomodulatory effects beneficial to IBD amelioration (IL-10 or α-MSH) are capable of stronger prevention/cure of UC-like colitis in mice than are WT strains, with effects presumably mediated through synergistic effects on various functions (Figure 1C).

## *Escherichia coli* Nissle 1917

*Escherichia coli* Nissle 1917 has no pathogenic factors (adhesion molecules, invasiveness, enterotoxin, cytotoxins, etc.). This strain’s genetics, physiology, and biological activities as a probiotics were largely characterized some time ago; as an alternative medicine (Mutaflor) for IBD and other GIT-related diseases, EcN currently serves as one of the most useful bacterial strains (104). In randomized controlled trials of UC remission maintenance, oral administration of EcN was as effective as treatment with mesalazine in preventing relapse of the disease (105–107). In studies using IBD model animals, EcN was proven to ameliorate colitis symptoms by regulation of the immune system and intestinal barrier function (108–111). In addition, the utility of this immunobiotic strain as a production platform for vaccines and pharmaceutics and as an intestinal delivery system continues to grow (112). Studies of gm-EcN that produces pathogenic bacteria/virus antigens (113–115) and immunomodulatory molecules such as cytokines and proteins derived from parasites (24, 25) have been reported, and disease preventive/curative effects have been verified in animals.

In 2012, Gardlik et al. developed IL-10-secreting EcN and verified this strain’s anti-inflammatory effects using DSS colitis (25). Oral administration of IL10-secreting EcN was shown to improve inflammation parameters (reduced stool consistency, colon shortening, decreased oxidative and carbonyl stress), but these effects were of the same degree as obtained with WT-EcN or IL-10-secreting MG1363. In 2014, EcN that secretes a protease inhibitor protein derived from nematodes (AvCys) was created (24). AvCys’ immune-regulatory action is mediated mainly by targeting macrophages, and this inhibitory protein exhibits anti-inflammatory action in murine models of IBD and allergies (116–119). Oral administration of AvCys-secreting EcN (EcN-AvCys) on alternate days attenuated DSS colitis by beneficial regulation of the immune system in the inflamed colon (regulation of the proportion and function of pro-inflammatory macrophages, increase in the proportion of FoxP3^+^ Treg cells, and decrease in inflammatory cytokines and chemokines). In addition, in experiments using pigs (whose GITs closely resemble those of humans), oral administration of EcN-AvCys on alternate days to post-weaning piglets reduced spontaneous colon inflammation. Interestingly, the results of that study suggested that EcN-AvCys ameliorates inflammation in this piglet model by improving intestinal barrier function rather than by regulating the intestinal immune system. WT-EcN shows some benefits in ameliorating murine intestinal inflammation, inducing Treg cells, and increasing transepithelial resistance in a culture of a human colonic epithelial cell strain, but the efficacies were significantly milder than those obtained with EcN-AvCys.

## Conclusion and Future Perspectives

Site-directed delivery of proteins that exhibit anti-inflammatory effects using gm-immunobiotics is extremely attractive as an effective preventive/curative strategy for IBD (Figure 1). A series of studies using IL-10-secreting *Lc. lactis*, ranging from basic to clinical, established a milestone by indicating the effectiveness and the feasibility of clinical application of this concept. Subsequently, gm-*Lc. lactis* strains that efficiently produce cytokines, enzymes, and protease inhibitors with a range of anti-inflammatory properties have been developed, and anti-inflammatory properties of these strains have been verified using rodent models of IBD (Table 1; Table S1 in Supplementary Material). Recent research into intestinal delivery of serine protease inhibitors and IL-27 has shown that these strains provide markedly more beneficial amelioration of murine intestinal inflammation than do strains that deliver IL-10. In addition, the research strongly implies that MG1363 and its derivatives do not have any negative impact on GIT inflammation or health maintenance, regardless of whether the strains are WT or recombinant. It may therefore be concluded that *Lc. lactis* is the bacterium that holds the most promise as a delivery agent for proteins with IBD therapeutic potential. In addition, work has also advanced to verify the potential for application of immunobiotics in this strategy. Interestingly, these studies show marked amelioration of GIT inflammation in animals as a result of the synergy between the immunoregulatory effects of the immunobiotic bacterium itself and the anti-inflammatory effects of the delivered RPs (Figure 1C; Table 1; Table S1 in Supplementary Material). This observation implies that the strategy of using immunobiotics is an effective means toward the development of IBD therapeutics with greater efficacy. For future work, it would be desirable to carry out comparative investigations of the therapeutic effects on GIT inflammation of different gm-strains that produce the same RP.

Clinical trials that include verification of safety and efficacy will be essential for developing gm-immunobiotics as therapeutic drugs for IBD. To date, there have been no findings that demonstrate any danger in the use of gm-probiotics including gm-immunobiotics. At the same time, there is little evidence to prove the safety of these agents in clinical use, and it remains possible that gm-probiotic organisms may be spread into the environment. Thus, there is some skepticism regarding the use of these agents. However, two clinical studies using IL-10-secreting *Lc. lactis* have demonstrated tremendous breakthroughs (59, 120, 121). In addition, in a recent phase 1b trial, oral administration of AG013 (an oral rinse containing trefoil factor 1-secreting MG1363 as the main component) was shown to be safe and well tolerated in cancer patients while also exhibiting efficacy against oral mucositis (122). Guidelines toward clinical use of gm-*Lc. lactis* have been proposed (123), and the feasibility of the clinical application of gm-*Lc. lactis* is strongly implied. With other probiotics, aspects such as the time for passage through the GIT, establishment in the GIT, health benefits, or the danger of side effects will differ from those of *Lc. lactis*, so safety evaluations will be needed and biological containment strategies will have to be developed. The establishment of effective gm-immunobiotics for prevention and treatment of IBD is near at hand, and it is to be hoped that this strategy will be facilitated by advances in the scientific understanding of gene recombination techniques in the future.

## Author Contributions

SS and TS conceived, designed, and wrote the manuscript.

## Conflict of Interest Statement

The authors declare that the research was conducted in the absence of any commercial or financial relationships that could be construed as a potential conflict of interest. The reviewer SA and handling Editor declared their shared affiliation, and the handling Editor states that the process nevertheless met the standards of a fair and objective review.
