# Supplementary material for: Applications of Genetically Modified Immunobiotics with High Immunoregulatory Capacity for Treatment of Inflammatory Bowel Diseases
Source: Front Immunol. 2017 Jan 25;8:22. doi: 10.3389/fimmu.2017.00022 (PMC5263139; doi:10.3389/fimmu.2017.00022)
Supplement: Supplementary file 1 [file Table_1.DOCX]

*Supplementary Material*

**Applications of genetically modified immunobiotics with high immunoregulatory capacity for treatment of inflammatory bowel diseases**

Suguru Shigemori, Takeshi Shimosato*

***Correspondence**:

Takeshi Shimosato

shimot@shinshu-u.ac.jp

**Supplementary Table 1. Pre-clinical evidences showing beneficial effects of gm-probiotics in treatment of GIT inflammation.**

| **Strains** | **Recombinant Protein** | **Disease model** | **Outcome** | **Efficacy** | **Potential mechanisms** | **Ref.** |
| --- | --- | --- | --- | --- | --- | --- |
| ***Lactococcus lactis*** | | | | | | |
| MG1363 | Anti-TNF Nanobody | mDCC, m*IL-10^-/-^* | Reduction in HS, and IM (MPO) | CC = VC < MG1363-IL-10 = Object (mDCA)  CC = Oral/Systemic anti-TNF Nanobody = VC = MG1363-IL-10 < Object (m*IL-10^-/-^*) | Neutralization of TNF | (1) |
| MG1363  /NZ9000 | IL-10 | mDAC, mTAC, m*IL-10^-/-^* | Reduction in MS, HS, and IM (MPO, Cox-2, SAA)  Modulation of P/AICy | CC = WT/VC < Objects | Immunomodulation | (2-6) |
| MG1363 | IL-27 | mTTC, mDAC | Reduction in Mo, MS, and HS  Modulation of P/AICy, and PTc | CC = Systemic IL-27 = VC < Object  MG1363-IL-10 < Object | Immunomodulation | (7) |
| MG1363 | LcrV | mDAC, mTAC | Reduction in MS, HS, CS, and IM (MPO, PICy, Cox-2, SAA) | CC = WT < MG1363-IL-10 = Object | Immunomodulation | (3) |
| MG1363 | MAM | mDNAC | Reduction in MS, and IM (PICy) | VC < Object | Immunomodulation | (8) |
| MG1363 | TSLP | mDAC | Reduction in MS, HS, and IM (PICy)  Increase in Treg | WT < Object | Immunomodulation | (9) |
| MG1363 | TTFs | mDAC, m*IL-10^-/-^* | Reduction in Mo, MS, HS, and IM (MPO)  Induction of Ptgs2 expression | CC = Oral/Rectal TTF = VC = MG1363-IL-10 < Objects | Promotion of wound healing in intestinal mucosa | (10) |
| NCDO 2118 | 15-LOX-1 | mTAC | Reduction in MS, HS, LMT | CC ≤ WT ≤ Object | Reduction in oxidative stress | (11) |
| NZ3900 | Cathelicidin | mDAC | Reduction in MS, HS, CS, Ap, FMP, and IM (MPO, PICy, MDA) | CC = VC ≤ SASP ≤ Object | Promotion of wound healing in intestinal mucosa | (12, 13) |
| NZ9000 | Elafin/SLPI | mDAC, mDCC, mTTC, hIEC | Reduction in MS, HS, CT, IIP, and IM (PL, MPO, PICy, PIL) | CC ≤ WT < *L. lactis* IL-10/TGF-β < Objects | Reduction in elastolytic activity | (2, 14) |
| NZ9000 | HO-1 | mDAC | Reduction in MS, HS, and CS  Modulation of P/AICy | CC = VC < Object | Immunomodulation | (15) |
| NZ9000 | IGF-I | mDAC | Reduction in HS, CS, and IM (MPO, DAO)  Increase in occludin | CC ≤ VC ≤ Object | Improvement of intestinal barrier function | (16) |
| NZ9800 | SOD | rTAC | Reduction in MS, HS, and IM (MPO, NT) | CC < WT = Object | Reduction in oxidative stress | (17) |
| ***Lactobacillus*** | | | | | | |
| *casei* BL23 | Cat/SOD | mDAC, mTAC | Reduction in MS, HS, and LMT  Modulation of P/AICy | CC ≤ WT/VC < Objects | Reduction in oxidative stress  Immunomodulation | (18-20) |
| *casei* BLS | α-MSH | mDAC | Reduction in Mo, MS, HS, CS, and IM (MPO, NF-κB)  Modulation of P/AICy | CC ≤ WT < Object | Immunomodulation | (21) |
| *casei* CECT 5276 | IL-10 | mDAC | Reduction in MS, HS, CS, and IM (NF-κB)  Modulation of P/AICy | 5-ASA  ≤ WT + 5-ASA  < Object + 5-ASA | Immunomodulation | (22) |
| *fermentum* I5007 | Cat | mDAC | Reduction in HS, IM (NF-κB, MPO, LP)  Increase in *Lb* and *Bi* in colon | CC = VC < V_E_ = Object | Reduction in oxidative stress | (23) |
| *fermentum* I5007 | SOD | mTAC | Reduction in MS, HS, IM (NF-κB, MPO, LP) | CC ≤ WT ≤ Object | Reduction in oxidative stress | (24) |
| *gasseri* NCK 334 | SOD | m*IL-10^-/-^* | Reduction in HS, IM (MPO, Cox-2)  Modulation of Ao | CC ≤ VC ≤ Object | Reduction in oxidative stress | (25) |
| *plantarum* NCIMB 8826 Int-1 | SOD | rTAC | Reduction in MS, HS, and IM (MPO, NT) | CC = WT < Object | Reduction in oxidative stress | (17) |
| **Others** | | | | | | |
| *B. longum* NCC2705 | IL-10 | mDAC | Reduction in Mo, MS, HS, CS and IM (MPO, NF-κB)  Modulation of PTc, and P/AICy | CC < WT/VC < Object | Immunomodulation | (26, 27) |
| *B. longum* HB15 | α-MSH | rDAC | Reduction in HS, and IM (MPO, NO)  Modulation of P/AIC  Increase in *Bi* in colon | CC ≤ WT < Object | Immunomodulation | (28) |
| *B. longum* HB25 | α-MSH | mDAC | Reduction in HS, and IM (MPO)  Modulation of P/AICy | CC = VC < Object | Immunomodulation | (29) |
| EcN | AvCys | mDAC, pPWD, hIEC | Reduction in MS, HS, CS, IIP, and IM (PIM, PICh, PICy)  Increase in Treg, TER | CC ≤ WT < Object | Immunomodulation  Improvement of intestinal barrier function | (30) |
| EcN | IL-10 | mDAC | Reduction in MS, CS, and IM (MDA, Fr) | CC ≤ ER2738 ≤ WT, MG1363-IL-10, Object | Immunomodulation | (31) |
| *S. thermophilus* CRL807 | Cat/SOD | mTAC | Reduction in Mo, MS, HS, and LMT  Modulation of CPIc | CC < WT < Objects | Reduction in oxidative stress  Immunomodulation | (32) |

Ref.: references, MG1363/NZ3900/NZ9000/NZ9800: *Lactococcus lactis* subsp. *cremoris* MG1363/NZ3900/NZ9000/NZ9800, NCDO 2118: *Lactococcus lactis* subsp. *lactis* NCDO 2118, *B. longum*: *Bifidobacterium longum*, EcN: *Escherichia coli* Nissle 1917, *S. thermophilus*: *Streptococcus salivarius* subsp. *thermophilus*, TNF: tumor necrosis factor, IL-10: interleukin 10, IL-27: interleukin 27, LcrV: low calcium response V antigen from *Yersinia* *pseudotuberculosis*, MAM: anti-inflammatory protein from *Faecalibacterium prausnitzii*, TSLP: thymic stromal lymphopoietin, TTFs: trefoil factors, 15-LOX-1: 15-lipoxygenase-1, SLPI: secretory leukocyte protease inhibitor, HO-1: heme oxygenase-1, IGF-I: insulin-like growth factor I, SOD: superoxide dismutase, Cat: catalase, α-MSH: α-melanocyte-stimulating hormone, AvCys: cystatin from *Acanthocheilonema viteae*, mDCC: murine dextran sulfate sodium-induced chronic colitis, m*IL-10^-/-^*: spontaneous colitis in IL-10 deficient mice, m/rDAC: murine/rat dextran sulfate sodium-induced acute colitis, m/rTAC: murine/rat 2,4,6-trinitrobenzene sulfonic acid-induced acute colitis, mTTC: murine T-cell transfer-induced enterocolitis, mDNAC: murine-dinitrobenzene sulfonic acid-induced acute colitis, hIEC, human intestinal epithelial cells, pPWD: porcine post-weaning diarrhea, HS: histological symptoms, IM: mediators of inflammation, MPO: myeloperoxidase activity, MS: macroscopic symptoms, Cox-2: cyclooxygenase-2 activity, SAA: serum amyloid A, P/AICy: pro-/anti-inflammatory cytokines, Mo: mortality, PTc: phenotypes of T-cell, CS: colon shortening, PICy: pro-inflammatory cytokines, Treg: regulatory T-cell, Ptgs2: prostaglandin-endoperoxide synthase 2, LMT: liver microbial translocation, Ap: apoptosis in colonic tissues, FMP: fecal microbiota populations, MDA: malonaldehyde activity, CT: colon thickening, IIP: intestinal epithelial permeability, PL: proteolytic activity, PIL: pro-inflammatory leukocytes, DAO: diamine oxidase activity, NT: nitrotyrosine, NF-κB: nuclear factor-κB, LP: lipid peroxidation, *Lb*: Lactobacilli, *Bi*: Bifidobacteria, Ao: antioxidants, NO: nitrogen monoxide, PIM: pro-inflammatory macrophages, PICh: pro-inflammatory chemokines, TER: transendothelial electrical resistance, Fr: fructosamine, CPIc: cytokine phenotypes of immune cells, CC: colitis control, VC: vector control, MG1363-IL-10: IL-10-secreting MG1363, WT: wild-type strain, SASP: sulfasalazine, NZ9000-IL-10/TGF-β: IL-10- or TGF-β-secreting NZ9000, 5-ASA: 5-aminosalicylate, V_E_: vitamin E, ER2738: *Escherichia coli* ER2738

**References**

1. Vandenbroucke K, de Haard H, Beirnaert E, Dreier T, Lauwereys M, Huyck L, et al. Orally administered *L. lactis* secreting an anti-TNF Nanobody demonstrate efficacy in chronic colitis. *Mucosal Immunol* (2010) **3**(1):49-56. doi: 10.1038/mi.2009.116

2. Bermudez-Humaran LG, Motta JP, Aubry C, Kharrat P, Rous-Martin L, Sallenave JM, et al. Serine protease inhibitors protect better than IL-10 and TGF-beta anti-inflammatory cytokines against mouse colitis when delivered by recombinant lactococci. *Microb Cell Fact* (2015) **14**:26. doi: 10.1186/s12934-015-0198-4

3. Foligne B, Dessein R, Marceau M, Poiret S, Chamaillard M, Pot B, et al. Prevention and treatment of colitis with *Lactococcus lactis* secreting the immunomodulatory *Yersinia* LcrV protein. *Gastroenterology* (2007) **133**(3):862-74. doi: 10.1053/j.gastro.2007.06.018

4. Steidler L, Hans W, Schotte L, Neirynck S, Obermeier F, Falk W, et al. Treatment of murine colitis by *Lactococcus lactis* secreting interleukin-10. *Science* (2000) **289**(5483):1352-5.

5. Waeytens A, Ferdinande L, Neirynck S, Rottiers P, De Vos M, Steidler L, et al. Paracellular entry of interleukin-10 producing *Lactococcus lactis* in inflamed intestinal mucosa in mice. *Inflamm Bowel Dis* (2008) **14**(4):471-9. doi: 10.1002/ibd.20346

6. del Carmen S, Martin Rosique R, Saraiva T, Zurita-Turk M, Miyoshi A, Azevedo V, et al. Protective effects of lactococci strains delivering either IL-10 protein or cDNA in a TNBS-induced chronic colitis model. *J Clin Gastroenterol* (2014) **48 Suppl 1**:S12-7. doi: 10.1097/mcg.0000000000000235

7. Hanson ML, Hixon JA, Li W, Felber BK, Anver MR, Stewart CA, et al. Oral delivery of IL-27 recombinant bacteria attenuates immune colitis in mice. *Gastroenterology* (2014) **146**(1):210-21.e13. doi: 10.1053/j.gastro.2013.09.060

8. Quevrain E, Maubert MA, Michon C, Chain F, Marquant R, Tailhades J, et al. Identification of an anti-inflammatory protein from *Faecalibacterium prausnitzii*, a commensal bacterium deficient in Crohn's disease. *Gut* (2016) **65**(3):415-25. doi: 10.1136/gutjnl-2014-307649

9. Aubry C, Michon C, Chain F, Chvatchenko Y, Goffin L, Zimmerli SC, et al. Protective effect of TSLP delivered at the gut mucosa level by recombinant lactic acid bacteria in DSS-induced colitis mouse model. *Microb Cell Fact* (2015) **14**:176. doi: 10.1186/s12934-015-0367-5

10. Vandenbroucke K, Hans W, Van Huysse J, Neirynck S, Demetter P, Remaut E, et al. Active delivery of trefoil factors by genetically modified *Lactococcus lactis* prevents and heals acute colitis in mice. *Gastroenterology* (2004) **127**(2):502-13.

11. Saraiva TD, Morais K, Pereira VB, de Azevedo M, Rocha CS, Prosperi CC, et al. Milk fermented with a 15-lipoxygenase-1-producing *Lactococcus lactis* alleviates symptoms of colitis in a murine model. *Curr Pharm Biotechnol* (2015) **16**(5):424-9.

12. Wong CC, Zhang L, Wu WK, Shen J, Chan RL, Lu L, et al. Cathelicidin-Encoding *Lactococcus lactis* Promotes Mucosal Repair in Murine Experimental Colitis. *J Gastroenterol Hepatol* (2016). doi: 10.1111/jgh.13499

13. Wong CC, Zhang L, Li ZJ, Wu WK, Ren SX, Chen YC, et al. Protective effects of cathelicidin-encoding *Lactococcus lactis* in murine ulcerative colitis. *J Gastroenterol Hepatol* (2012) **27**(7):1205-12. doi: 10.1111/j.1440-1746.2012.07158.x

14. Motta JP, Bermudez-Humaran LG, Deraison C, Martin L, Rolland C, Rousset P, et al. Food-grade bacteria expressing elafin protect against inflammation and restore colon homeostasis. *Sci Transl Med* (2012) **4**(158):158ra44. doi: 10.1126/scitranslmed.3004212

15. Shigemori S, Watanabe T, Kudoh K, Ihara M, Nigar S, Yamamoto Y, et al. Oral delivery of *Lactococcus lactis* that secretes bioactive heme oxygenase-1 alleviates development of acute colitis in mice. *Microb Cell Fact* (2015) **14**:189. doi: 10.1186/s12934-015-0378-2

16. Liu S, Li Y, Deng B, Xu Z. Recombinant *Lactococcus lactis* expressing porcine insulin-like growth factor I ameliorates DSS-induced colitis in mice. *BMC Biotechnol* (2016) **16**:25. doi: 10.1186/s12896-016-0255-z

17. Han W, Mercenier A, Ait-Belgnaoui A, Pavan S, Lamine F, van S, II, et al. Improvement of an experimental colitis in rats by lactic acid bacteria producing superoxide dismutase. *Inflamm Bowel Dis* (2006) **12**(11):1044-52. doi: 10.1097/01.mib.0000235101.09231.9e

18. LeBlanc JG, del Carmen S, Miyoshi A, Azevedo V, Sesma F, Langella P, et al. Use of superoxide dismutase and catalase producing lactic acid bacteria in TNBS induced Crohn's disease in mice. *J Biotechnol* (2011) **151**(3):287-93. doi: 10.1016/j.jbiotec.2010.11.008

19. Rochat T, Bermudez-Humaran L, Gratadoux JJ, Fourage C, Hoebler C, Corthier G, et al. Anti-inflammatory effects of *Lactobacillus casei* BL23 producing or not a manganese-dependant catalase on DSS-induced colitis in mice. *Microb Cell Fact* (2007) **6**:22. doi: 10.1186/1475-2859-6-22

20. Watterlot L, Rochat T, Sokol H, Cherbuy C, Bouloufa I, Lefevre F, et al. Intragastric administration of a superoxide dismutase-producing recombinant *Lactobacillus casei* BL23 strain attenuates DSS colitis in mice. *Int J Food Microbiol* (2010) **144**(1):35-41. doi: 10.1016/j.ijfoodmicro.2010.03.037

21. Yoon SW, Lee CH, Kim JY, Kim JY, Sung MH, Poo H. *Lactobacillus casei* secreting α-MSH induces the therapeutic effect on DSS-induced acute colitis in Balb/c Mice. *J Microbiol Biotechnol* (2008) **18**(12):1975-83.

22. Qiu ZB, Chen J, Chen JJ, Rong L, Ding WQ, Yang HJ, et al. Effect of recombinant *Lactobacillus casei* expressing interleukin-10 in dextran sulfate sodium-induced colitis mice. *J Dig Dis* (2013) **14**(2):76-83. doi: 10.1111/1751-2980.12006

23. Zhang J, Liu H, Wang Q, Hou C, Thacker P, Qiao S. Expression of catalase in *Lactobacillus fermentum* and evaluation of its anti-oxidative properties in a dextran sodium sulfate induced mouse colitis model. *World J Microbiol Biotechnol* (2013) **29**(12):2293-301. doi: 10.1007/s11274-013-1395-0

24. Hou CL, Zhang J, Liu XT, Liu H, Zeng XF, Qiao SY. Superoxide dismutase recombinant *Lactobacillus fermentum* ameliorates intestinal oxidative stress through inhibiting NF-kappaB activation in a trinitrobenzene sulphonic acid-induced colitis mouse model. *J Appl Microbiol* (2014) **116**(6):1621-31. doi: 10.1111/jam.12461

25. Carroll IM, Andrus JM, Bruno-Barcena JM, Klaenhammer TR, Hassan HM, Threadgill DS. Anti-inflammatory properties of *Lactobacillus gasseri* expressing manganese superoxide dismutase using the interleukin 10-deficient mouse model of colitis. *Am J Physiol Gastrointest Liver Physiol* (2007) **293**(4):G729-38. doi: 10.1152/ajpgi.00132.2007

26. Yao J, Wang JY, Lai MG, Li YX, Zhu HM, Shi RY, et al. Treatment of mice with dextran sulfate sodium-induced colitis with human interleukin 10 secreted by transformed *Bifidobacterium longum*. *Mol Pharm* (2011) **8**(2):488-97. doi: 10.1021/mp100331r

27. Zhang D, Wei C, Yao J, Cai X, Wang L. Interleukin-10 gene-carrying bifidobacteria ameliorate murine ulcerative colitis by regulating regulatory T cell/T helper 17 cell pathway. *Exp Biol Med (Maywood)* (2015) **240**(12):1622-9. doi: 10.1177/1535370215584901

28. Wei P, Yang Y, Ding Q, Li X, Sun H, Liu Z, et al. Oral delivery of *Bifidobacterium longum* expressing α-melanocyte-stimulating hormone to combat ulcerative colitis. *J Med Microbiol* (2016) **65**(2):160-8. doi: 10.1099/jmm.0.000197

29. Wei P, Yang Y, Liu Z, Huang J, Gong Y, Sun H. Oral *Bifidobacterium longum* expressing α-melanocyte-stimulating hormone to fight experimental colitis. *Drug Deliv* (2016) **23**(6):2058-64. doi: 10.3109/10717544.2015.1122672

30. Whelan RA, Rausch S, Ebner F, Gunzel D, Richter JF, Hering NA, et al. A transgenic probiotic secreting a parasite immunomodulator for site-directed treatment of gut inflammation. *Mol Ther* (2014) **22**(10):1730-40. doi: 10.1038/mt.2014.125

31. Gardlik R, Palffy R, Celec P. Recombinant probiotic therapy in experimental colitis in mice. *Folia Biol (Praha)* (2012) **58**(6):238-45.

32. Del Carmen S, de Moreno de LeBlanc A, Martin R, Chain F, Langella P, Bermudez-Humaran LG, et al. Genetically engineered immunomodulatory *Streptococcus thermophilus* strains producing antioxidant enzymes exhibit enhanced anti-inflammatory activities. *Appl Environ Microbiol* (2014) **80**(3):869-77. doi: 10.1128/aem.03296-13
